# Autosomal recessive primary microcephaly in sibs in time of Zika epidemic: a Case Report

**DOI:** 10.3389/fgene.2025.1565296

**Published:** 2025-05-22

**Authors:** Julia V. Almeida, Bianca Barbosa Abdala, Natana Chaves Rabelo, Maria Eduarda Gomes, Elenice Ferreira Bastos, Juan Clinton Llerena, Sayonara Gonzalez

**Affiliations:** ^1^ Medical Genetics Center, Instituto Nacional Fernandes Figueira, Fiocruz, Rio de Janeiro, Brazil; ^2^ Genomic Medicine Laboratory, Instituto Nacional Fernandes Figueira, Fiocruz, Rio de Janeiro, Brazil

**Keywords:** microcephaly, MCPH, ASPM, congenital Zika syndrome, autosomal recessive primary microcephaly

## Abstract

Case report of two siblings, born to consanguineous parents, with congenital microcephaly secondary to a pathogenic homozygous *ASPM* gene variant. The proband was born during the Zika virus epidemic with a prenatal history of maternal exanthematous disease. Genetic diagnosis was made after the birth of the sibling, born with a similar condition. Next-generation sequencing enables a definitive diagnosis in cases of microcephaly, and genetic diagnosis should be pursued even when the patient history points to a possible, but not definite, environmental cause. Conclusive genetic diagnosis allows for precise and timely family planning and counseling.

## 1 Introduction

Congenital microcephaly is a malformation of cortical development that has several different causes. It is characterized by head circumference at birth (HC) that is more than two standard deviations (SD) below the mean for age and sex (Z below −2) (Woods and Parker, 2013). Approximately 29% of cases have a genetic cause, with different patterns of inheritance. Autosomal recessive microcephalies are collectively called autosomal recessive primary microcephaly (MCPH) and represent approximately 8% of genetic microcephalies. One study featuring 680 patients with microcephaly found identifiable pre- or postnatal brain injury, such as birth complications or congenital infections, in 28.6%, while 28.5% had an identifiable genetic etiology (Von der Hagen et al., 2014).

During the Brazilian Zika epidemic, the incidence of congenital microcephaly increased nine-fold above predicted. New diagnostic criteria for this emerging disease had to be developed (OPAS, 2016). Healthcare workers also faced diagnostic challenges: while positive PCR results were reliable during acute infection, serological tests for past infection were often unreliable due to cross-reactivity with other mosquito-borne flaviviruses endemic to Brazil (Alvarado-Socarras et al., 2018; Chan et al., 2022).

Here, we report the case of a patient born during the peak of the Zika virus congenital syndrome (CZS) epidemic (2015–2016). His mother presented an unidentified but likely viral exanthematous infection during pregnancy, which, in the context of the epidemic and in the presence of congenital microcephaly, was sufficient to define the case as a probable infectious etiology. Initial genetic investigation and evaluation of the proband were inconclusive but incomplete. After the birth of an affected sibling 4 years later, the use of whole-exome sequencing (WES) led to the diagnosis of MCPH caused by a homozygous pathogenic *ASPM* variant.

MCPH may be caused by disruptions in a variety of mechanisms involved in the regulation of cerebral cortical development, particularly those involved in centriole biogenesis, DNA replication and repair, cytokinesis, and others. Two of the genes most associated with MCPH, *ASPM* and *WDR62* (related to MCPH5 and MCPH2, respectively), are part of a larger protein complex involving other MCPH genes and relating to centrosome function, integral to neuronal proliferation (Jayaraman et al., 2018).

Epidemiology-based diagnostic criteria faced with consanguinity or a positive family history of disease should be taken with a grain of salt as the correct etiological definition can strongly impact genetic counseling and the couple’s reproductive planning.

## 2 Case report

**FIGURE 1:**
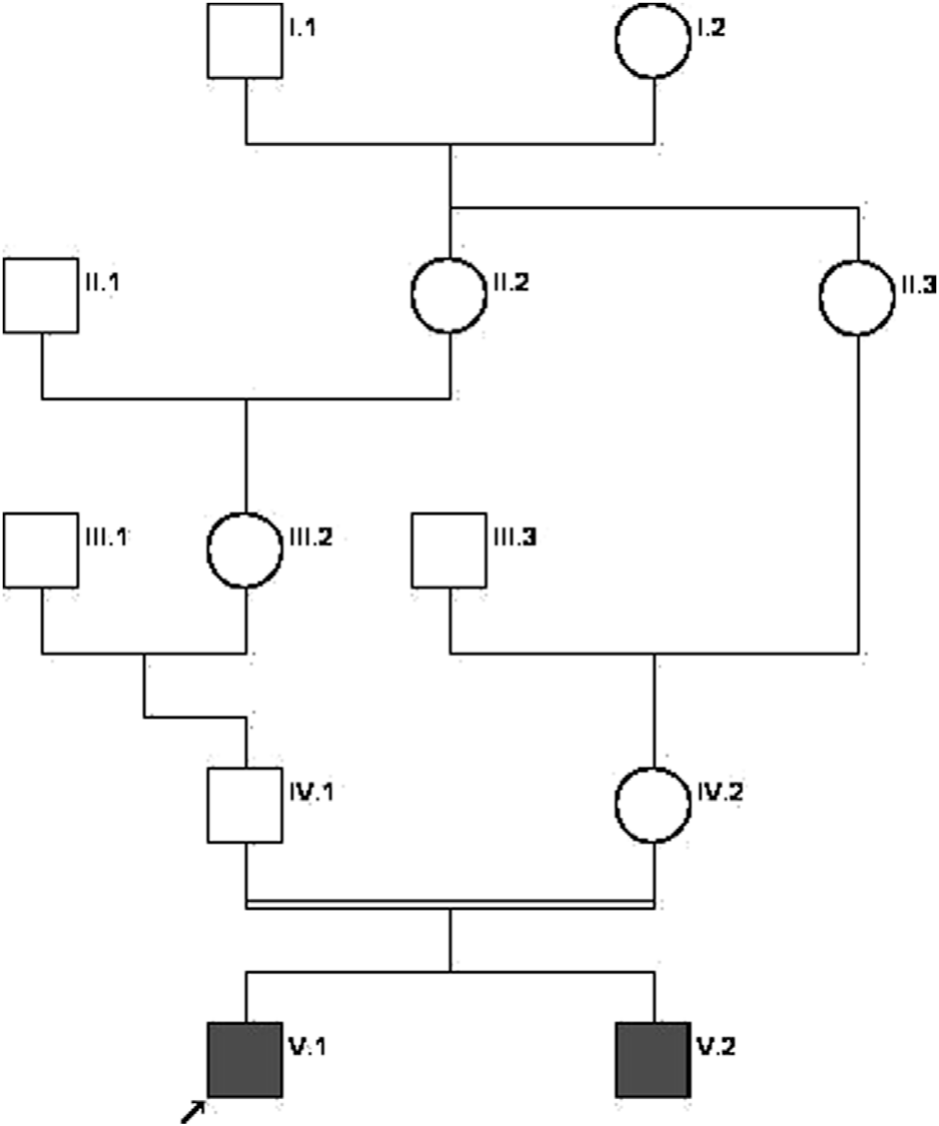
Family pedigree chart. The proband is indicated by the arrow. Affected individuals are shown in solid dark gray.

The male proband, the first child of distant consanguineous parents with no family history of genetic or neurological disease (Figure 1), was born with congenital microcephaly (HC 28 cm, Z score −3.46) in October 2015 during the Zika virus epidemic in Brazil (Pielnaa et al., 2020; OPAS, 2016). The mother had a history of skin rash and swollen lymph nodes in the 6th month of pregnancy, diagnosed as rubella at the time. After birth, the child was evaluated by infectious disease specialists, who referred him for genetic evaluation due to a history of consanguinity and for neurological follow-up. The first evaluation at the genetics clinic happened at 6 months of age. He exhibited an HC of 32 cm (Z score −8.59). The child presented hypertonia since birth and developmental delay (he smiled at 4 months but was unable to roll, babble, or sit with support). On examination, he had increased deep tendon reflexes, significant global hypertonia, excessive scalp skin, closed fontanelles, and a prominent occipital bone.

A Zika virus (ZIKV) PCR assay (at 3 months of age) was negative. Maternal serological investigation for toxoplasmosis had negative immunoglobulin G (IgG) titers in the first trimester, positive in the second trimester (9.48, normal values < 1.0), and again negative in the third. Second-trimester titers were considered false positives. Cytomegalovirus (CMV) IgG titers were positive with negative immunoglobulin M (IgM) titers throughout pregnancy. Rubella IgG titers were positive in both the first and third trimesters, with a five-fold increase in the third. Other tests for toxoplasmosis, rubella, cytomegalovirus, herpes simplex, and syphilis (TORCHS) were negative. ZIKV serological tests and PCR were unavailable during the proband’s gestational period.

A neonatal cranial ultrasound showed small echogenic foci in the nucleocapsular and subcortical regions, possibly corresponding to calcifications, with thinned cerebral parenchyma and poorly defined sulci. An MRI (at 1 month) revealed craniofacial disproportion, diffuse cortical sulcus accentuation, and intact nucleocapsular regions. A CT scan (at 2 months) showed wide gyral patterns, reduced volume of the frontal lobes, and an image suggestive of closed-lip schizencephaly in the right parietal region without calcifications. The abdominal ultrasound and echocardiogram were normal. The EEG was normal.

The proband was initially diagnosed with microcephaly, likely caused by congenital infection. Despite the probable infectious cause, a genetic investigation was initiated due to the history of consanguinity. Resources at this point only allowed a limited investigation. Karyotype analysis showed a normal male pattern (46, XY). NGS panels were not available due to financial limitations (both institutional and familial), making it impossible to definitively confirm or exclude either the infectious or genetic etiology. At that point, most of the research resources available were focused on dealing with the novelty of the Zika virus epidemic.

When the proband was 4 years old, his brother was born. He had a history of intrauterine growth restriction and congenital microcephaly. The brother was first evaluated by the neurology clinic at 8 months. He had severe microcephaly (Z score −9.88), global developmental delay, hypertonia, and hyperreflexia. The family was again referred for genetic evaluation. The brother had positive IgG ZIKV serology and negative IgM (at 1 year and 7 months). A CT scan (11 months) showed diffuse thinning of the cerebral parenchyma with prominence of the cortical sulci, without ventricular dilation, and significant craniofacial disproportion, occipital protrusion, and closed anterior fontanel and sutures. His karyotype analysis also showed a normal male pattern (46, XY, 13ps+).

Commercial multigene panels with restricted gene selection available at the time were negative.

In 2022, when the proband was 7 years old, the Reference Service for Rare Diseases of our institute (SRRD/IFF/Fiocruz) began using WES to test undiagnosed patients after completing a pilot study on genetically heterogeneous conditions (Rabelo et al., 2022). Genomic DNA was isolated from peripheral blood using a PureLink^®^ Genomic DNA MiniKit (Invitrogen-ThermoFisher). Library construction used Twist Exome 2.0 (Twist Bioscience), and sequencing was performed in paired-end mode on NovaSeq 6000 (Illumina).

Data processing was done with the Varstation tool (Genesis Genomics) and included BWA-MEM, Picard MergeBamAlignment, Picard MarkDuplicates, GATK BaseRecalibrator, and ApplyBQSR for mapping; GATK HaplotypeCaller for variant calling; and SNV/Indel Annotation for annotation. WES data had an average depth of 160x, with 99.97% of target regions at ≥10x depth and 97.57% with ≥Q30 quality.

Variant filtering and prioritization were performed by first removing variants of low-quality and with insufficient depth. Filtered was based on minor allele frequency using GnomAD, focusing on rare or novel variants. *In silico* tools were used to assess variants’ functional impact, prioritizing those with predicted damaging effects. A homozygous c.9697C>T variant was found in exon 24 of the *ASPM* gene, with a coverage depth of 218x (VAF = 1). It creates a premature termination codon (p.Arg3233*), probably resulting in nonsense-mediated decay (NMD), because the premature termination codon (PTC) is not occurring in the 3’ most exon or the 3’-most 50 bp of the penultimate exon of the canonical transcript (NM_018136.5). The variant is rare in population database (GnomAD), where it appears only in the heterozygous state. ClinVar contains an entry for this variant (Variation ID: 21634). It has also been identified in compound heterozygosity with a pathogenic variant in two siblings with MCPH (MCPH5) (Moriwaki et al., 2019). Based on these findings, this variant was classified as pathogenic according to ACMG/AMP criteria (PVS1, PM2_Supporting, and PM3).

## 3 Discussion

Congenital microcephaly is defined as an HC Z score below −2 (Woods CG and Parker A, 2013). In typical epidemiological situations, it is estimated that congenital microcephaly occurs in 0.5% of live births, with the incidence of secondary microcephaly (postnatal onset) being even higher (O’Kane and Begg, 2019). Environmental causes of congenital microcephaly include fetal exposure to teratogens or vascular and infectious complications during pregnancy (Sadler T, 2019; Von der Hagen et al., 2014).

The Zika virus epidemic profoundly altered the known statistics of microcephaly. Between 2000 and 2014, the mean annual average of microcephaly cases was 5 per 100,000 live births (with a mean of 164 new cases per year, with an SD of 15). In October 2015, the state of Pernambuco reported the first 26 cases of microcephaly associated with congenital ZIKV infection in Brazil. In the following months, the incidence of new cases of congenital microcephaly in Brazil increased ten-fold above the expected for the population (54.6 cases per 100,000 live births), with the Northeast Region reaching an even higher number of cases (139 per 100,000 live births). By the end of 2018, nearly 3,000 new cases of congenital microcephaly had been registered (Crisanto-López et al., 2023; Marinho et al., 2016).

In November 2015, with the rising number of congenital microcephaly cases in association with ZIKV infection, Brazil declared a public health emergency (Brazilian Ministry of Health, 2015). The WHO followed suit in February 2016. This syndrome results from the direct action of the virus, with a marked reduction in brain volume, leading to extremely severe microcephaly, cranial morphology abnormalities, significant neurological impairment, and functional changes. Brain injury is characterized by intracranial calcifications, enlarged ventricles, increased extra-axial cerebrospinal fluid spaces, cortical thinning, hypoplasia or agenesis of the corpus callosum, reduced myelination, cerebellar hypoplasia, and cortical development anomalies such as polymicrogyria, pachygyria, and agyria (Crisanto-López et al., 2023).

The reported proband met the Pan American Health Organization criteria for a “probable” case of congenital syndrome associated with Zika virus infection. A “confirmed” case would require laboratory evidence of ZIKV exposure, independent of the detection of other causal agents (OPAS, 2016.).

In a period marked by uncertainties brought about by the emergence of a new disease, with an abrupt and significant increase in cases, lack of reliable tests, and intense media coverage of the CZS epidemic, many families found themselves in vulnerable situations. This also led to the emergence of alliances and solidarity among families. These situations highlighted the needs of children with disabilities and their families, not only due to CZS but from many other causes, including rare diseases, who had long required support from public agencies (Melo et al., 2023).

Disorders of neuronal proliferation lead to a reduction in the number of neurons and final brain volume, known as microcephaly, which can be observed as early as 32 weeks of gestation. Primary microcephaly refers to those caused by the failure of genetic mechanisms involved in neuronal proliferation (O’Kane and Begg, 2019; Woods and Parker, 2013). In addition to the reduced brain volume, they are associated with varying severities of intellectual disability, epilepsy, cerebral palsy, and behavioral problems. Genetic microcephalies can occur in a non-syndromic fashion or are associated with genetic syndromes, and inheritance patterns are varied (Létard et al., 2018; O’Kane and Begg, 2019; Von der Hagen et al., 2014).

In a series of 194 patients with genetic microcephaly, only 17 (8.7%) were caused by autosomal recessive disease (Von der Hagen et al., 2014). There are more than 30 genes associated with MCPH, *ASPM* accounting for most identified cases (Sajid Hussain et al., 2013; Wu et al., 2023).

*ASPM*, like other MCPH genes, encodes centrosomal proteins and regulates symmetric cell division (Wu et al., 2023). The ASPM protein forms the centromere of the apical neuroprogenitor cells and is involved in the adequate positioning of the spindle pole during neurogenesis. *ASPM* loss-of-function leads to MCPH5 (OMIM # 608716) (Jayaraman et al., 2018; Létard et al., 2018). Our patient carried a homozygous nonsense variant. Likewise, other patients reported in the literature and online databases have been shown to carry mostly nonsense or frameshift mutations, leading either to early truncated nonfunctional proteins or absent protein synthesis due to NMD of the mutant mRNA (Sajid Hussain et al., 2013). As of March 2025, only three missense variants have been reported on ClinVar as likely pathogenic, as opposed to hundreds of nonsense/frameshift variants and 23 splice site mutations.

When studying patients with malformations of cortical development, it is important to discern patients with congenital infection and optimize the usage of resources, but common sense must prevail, and extra efforts must be made to differentiate probable from definitive cases of congenital etiology and proceed with molecular investigations accordingly.

Having a confirmed and definitive diagnosis provides an explanation and a sense of relief for parents, especially in cases of familial recurrence. Furthermore, the identification of a genetic pathogenic variant allows for prenatal testing in future pregnancies of this couple, as well as family planning using preimplantation genetic testing, if desired. Additionally, other family members can be offered the opportunity to be tested for carrier status.

This cautionary tale reinforces the importance of chasing a definitive genetic diagnosis as early as possible so we can provide the best possible advice and care to families and so they can make informed decisions with regard to their future.

## 4 Patient perspective

This study was approved by our institute’s ethics committee, and written informed consent from the patients’ parents was obtained for this publication. Genetic counseling provided parents with education on the matter and presented them with options going forward. At the time of the genetic testing results, the couple discussed here did not intend on having another natural pregnancy, and the costs involved in preimplantation tests and *in vitro* fertilization were a limiting factor for them.

## Data Availability

The data presented in the study have been submitted to the ClinVar repository, under Submission ID: SUB15311355.
